# Membrane Vesicles as Drug Delivery Systems: MISEV, In Vivo Fluorescence Imaging and Tracking, Specific Tissue Targeting, and Therapeutic Application in Diseases

**DOI:** 10.3390/pharmaceutics17121550

**Published:** 2025-11-30

**Authors:** Ying Qin, Hongda Zhuang, Xiaoyong Ren, Mengyi Lan, Shuoshuo Fan, Zhitao Qiu, Junfang Zhao, Yong Chen

**Affiliations:** 1 The MOE Basic Research and Innovation Center for the Targeted Therapeutics of Solid Tumors, School of Pharmacy, Jiangxi Medical College, Nanchang University, Nanchang 330031, China; yingqin0505@ncu.edu.cn (Y.Q.); hongdadazhuang@gmail.com (H.Z.); xy1005@email.ncu.edu.cn (X.R.); lanmengyi0811@163.com (M.L.); fanshuoshuo111@163.com (S.F.); 2 Institute for Advanced Study, Nanchang University, Nanchang 330031, China; 3 College of Life Sciences, Nanchang University, Nanchang 330031, China; m17607049985@163.com (Z.Q.); 15872335771@163.com (J.Z.)

**Keywords:** membrane vesicles, drug delivery systems (DDSs), fluorescence imaging and tracking, specific tissue targeting, therapeutic applications

## Abstract

In the last decade, notable developments have occurred regarding the application of membrane vesicles—encompassing extracellular vesicles (EVs, including exosomes, microvesicles, apoptotic bodies, and others), self-organized cellular-membrane-derived vesicles, and isolated cell-bound membrane vesicles, among others—as bioinspired drug delivery systems (DDSs). A collection of 10 papers on such advances was published in the Special Issue of Pharmaceutics entitled “Advances of membrane vesicles in drug delivery systems, 2nd Edition”. These papers investigate the Minimum Information for Studies of Extracellular Vesicles (MISEV), in vivo fluorescence imaging and tracking, in vivo specific tissue targeting, and the therapeutic application of membrane vesicles as DDSs in cancers, osteoarthritis, ocular disorders, intestinal disease, and kidney diseases. The present article briefly summarizes these related topics and provides novel insights into the research on membrane vesicles as DDSs.

## 1. Membrane Vesicles as Drug Delivery System (DDSs) Are Rapidly Developing

**Figure 1:**
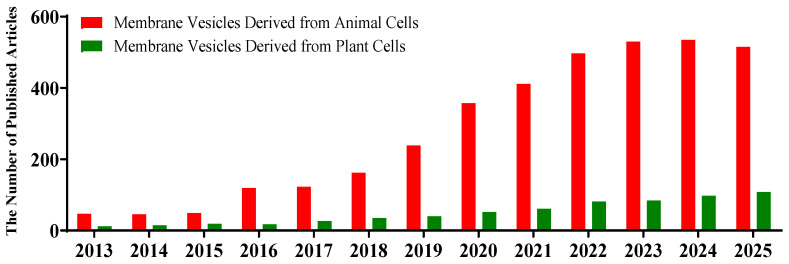
**The number of articles published each year on membrane vesicles from animal (red) and plant (green) cells as drug delivery systems (2013–2025).** The data were obtained from the Web of Science database by searching for article titles or the abstract field using the following keywords: ‘exosome’ or ‘apoptotic body’ or ‘cell-bound membrane vesicles’ or ‘microvesicle’ or ‘plant vesicles’ and ‘delivery’ (publication data are as of 19 October 2025).

Drug delivery systems (DDSs) regulate the absorption, distribution, metabolism and excretion of drugs in the body through specific carriers or technologies, in order to enhance therapeutic effects and reduce toxic side effects. They have been rapidly evolving towards precision and intelligence. Membrane vesicles are nanometer-sized particles with a double-layered membrane structure. Various types of membrane vesicles have been reported, including extracellular vesicles (EVs, including exosomes, microvesicles/microparticles, apoptotic bodies, etc.), cellular-membrane-derived vesicles reorganized after the isolation of cellular plasma membrane [1], and cell-bound membrane vesicles (CBMVs) isolated in situ from cell surfaces [2,3,4,5,6,7], among others. Due to their unique biological properties and the fast development of isolation/characterization techniques [8,9,10,11], membrane vesicles have shown great potential for application in the field of drug delivery, particularly after the 2013 Nobel Prize in Physiology or Medicine was awarded to Profs. James E. Rothman, Randy W. Schekman, and Thomas C. Südhof for discovering the mechanism that regulates the transport of intracellular vesicles. Membrane vesicles have diverse natural sources [12,13], including animal cells [14], plant cells [15], microorganisms [16], and biological fluids [9], among others. The application of variably derived EVs as drug delivery systems has developed rapidly in recent 10 years (Figure 1 shows the number of publications by year during 2013–2025 on drug delivery applications of membrane vesicles derived from animal cells and plant cells).

To closely follow the rapid development of this field, we recently organized a Special Issue entitled “Advances of membrane vesicles in drug delivery systems” in the journal *Pharmaceutics*. In the first edition of the Special Issue, 25 relevant papers were collected, mainly focusing on the source, preparation, modification, drug loading, and in vivo administration and biodistribution of membrane vesicles (mainly extracellular vesicles or exosomes and bacterial outer membrane vesicles), as well as their applications in the treatment of various diseases, including bone-related, gastrointestinal, organ transplant-related, cardiovascular, and neurodegenerative disorders, among others [17]. In the current, second edition of the Special Issue, 10 papers have been published, mainly focusing on in vivo fluorescence imaging and tracking, in vivo specific tissue targeting, and therapeutic applications in cancers, osteoarthritis, ocular disorders, and intestinal and kidney diseases.

## 2. The Minimum Information for Studies of Extracellular Vesicles (MISEV)

Extracellular vesicles are the most widely studied type of membrane vesicles. To foster the development of extracellular vesicle research, the International Society for Extracellular Vesicles (ISEV) was established in 2011. Considering that membrane vesicles face a series of severe challenges, including low reproducibility of experimental results and inconsistent research quality, the Minimum Information for Studies of Extracellular Vesicles (MISEV2014) guideline was created in 2014, with subsequent evolutions in 2018 and 2023 (Zhang et al. conducted a summary of the ten-year evolution of MISEV [18]). In the latest version of MISEV (i.e., MISEV2023), more extracellular particles were recommended in the nomenclature, including EV mimetics and artificial cell-derived vesicles (ACDVs), alongside traditional extracellular vesicles (exosomes, microvesicles, and apoptotic bodies), non-vesicular extracellular particles (NVEPs), and synthetic vesicles (SVs). MISEV2023 elaborates on the nomenclature, collection and pre-processing, characterization, release and cell uptake, functional activities, and in vivo studies of extracellular vesicles [19,20]. Common methods include labeling endogenous EVs or marking exogenous EVs with fluorescence or bioluminescence. When detecting and tracking endogenous and exogenous EVs, it is necessary to take into account the limitations of the specific sensitivity and spatial resolution of the technology. However, in vivo specific tissue targeting of extracellular vesicles receives less consideration in MISEV2023.

## 3. In Vivo Fluorescence Imaging and Tracking of Membrane Vesicles as DDSs

Membrane vesicles, especially small extracellular vesicles (sEVs), are promising natural nanocarriers for drug delivery and diagnostics due to their biocompatibility, low immunogenicity, and ability to cross biological barriers. A major hurdle in their clinical translation, however, is the precise monitoring of their in vivo journey, which currently depends predominantly on fluorescence imaging techniques. Several studies in this Special Issue performed in vivo fluorescence imaging and tracking of membrane vesicles as DDSs.

The research conducted by Krishnan et al. [21] represents significant progress in the field of targeted nanomedicine. They developed a promising drug delivery system that can overcome the biological barriers that have long hindered the effective treatment of peripheral nerve lesions. This advancement was achieved through the use of the IVIS^®^ Spectrum system, which can obtain both in vivo and in vitro imaging data, clearly demonstrating the efficacy of targeted drug delivery.

Using sEVs derived from human umbilical cord mesenchymal stem cells, HEK293T cells, and gastric cancer cells, Chen et al. [22] labeled these vesicles with either the lipophilic dye PKH26 (visible spectrum) or amine-modified PbS quantum dots (NIR-II region). They revealed that PKH26-labeled sEVs exhibited significant tumor accumulation, consistent with the expected enhanced permeability and retention effect and active targeting mechanisms. In contrast, PbS QD-labeled sEVs were predominantly sequestered in the liver and spleen, with minimal tumor signal—a divergence attributed to the differential dye leakage kinetics and possible interactions with the mononuclear phagocyte system. Moreover, the study highlights the benefits of NIR-II imaging—such as enhanced tissue penetration and minimal autofluorescence—confirming its value for real-time in vivo tracking. However, the rapid leakage of PbS QDs underscores a critical trade-off between image quality and labeling reliability. These insights will help refine EV engineering standards and advance EV-based therapies for cancer and other diseases.

**Figure 2:**
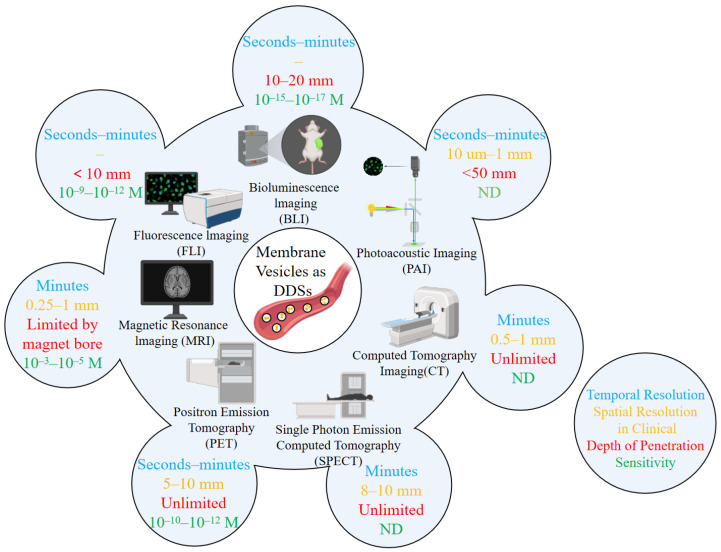
**Main technologies for in vivo imaging/tracking of EVs or membrane vesicles as drug delivery systems (the diagram is modified from Petroni et al. [26]).** The blue text represents “Temporal Resolution”, the orange text represents “Spatial Resolution in Clinical”, the red text represents “Depth of Penetration”, and the green text represents “Sensitivity”.

Molecular imaging technology can visualize biological and pathological processes at cell, molecular or genetic levels. It mainly includes magnetic resonance imaging (MRI) [23,24] and optical imaging [25]. Similarly, imaging techniques can applied to track and visualize membrane vesicles. Petroni et al. [26] investigated imaging techniques for extracellular EVs. With its non-invasive advantage, molecular imaging (MI) technology enables real-time visualization of the behavioral trajectory of extracellular EVs in living organisms. It provides indispensable data support for revealing the mechanisms of cell-to-cell communication mediated by EVs and promoting their clinical translation. Currently, the mainstream molecular imaging techniques applied to EV tracking are as follows: fluorescence imaging (FLI), Bioluminescence Imaging (BLI), Nuclear Imaging (PET/SPECT), magnetic resonance imaging (MRI), Photoacoustic Imaging (PAI), Computed Tomography Imaging (CT), and Multimodal Imaging. The application of these techniques can provide more comprehensive information for the study of EV behavior in vivo (Figure 2).

## 4. In Vivo Specific Tissue Targeting of Membrane Vesicles as DDSs

Membrane vesicles can achieve in vivo specific tissue targeting through the combination of “innate targeting potential exploration” and “acquired targeting modification”, enabling the carrier to precisely identify the target tissue. This is the key for it to progress from basic research to clinical application.

Krishnan et al. [21] developed a novel delivery system for targeted drug administration to peripheral nerves. The system utilized red blood cell-derived exosomes as carriers, functionalized with tetanus toxin-C fragment (TTC) on their surface via click chemistry as a targeting moiety. In vivo experiments demonstrated that intramuscularly injected TTC-functionalized exosomes effectively accumulated in target tissues, including the sciatic nerve and neuromuscular junctions, through retrograde axonal transport. In contrast, unmodified exosomes primarily remained at the injection site. Significantly lower signals detected in major organs such as the liver and spleen confirmed the high targeting specificity and limited systemic exposure of this delivery platform.

Zhao et al. [27] systematically and elaborately expounded the application of the membrane vesicle-based drug delivery system (MV-DDSs) in the treatment of inflammatory bowel disease (IBD). These systems achieve targeted accumulation in inflamed intestinal tissues through inherent tropism or engineered strategies such as surface ligand conjugation and stimulus-responsive design. MV-DDSs exert their therapeutic effects through multi-mechanistic actions, including anti-inflammatory signaling suppression, oxidative stress alleviation, intestinal barrier repair, gut microbiota modulation, and regulation of programmed cell death pathways. The evidence demonstrates that MV-DDSs represents a potent and targeted therapeutic strategy for IBD.

In recent years, it has been recognized that the tumor microenvironment plays a crucial role in the development and progression of cancer. This shift has led to the exploration of new treatment strategies targeting the tumor microenvironment [28]. The core of breast cancer targeted therapy revolves around the tumor microenvironment, including strategies such as immune checkpoint inhibitors, the regulation of tumor-associated macrophages, aromatase inhibitors, anti-angiogenic drugs, and intervention in signaling pathway targets. The targeted application of exosomes is an emerging direction. Engineered exosomes loaded with drugs/small interfering RNA (siRNA) target specific molecules (such as c-Met, MEK1) of subtypes such as TNBC, enhancing targeting and efficacy.

## 5. Therapeutic Application of Membrane Vesicles as DDSs in Diseases

**Figure 3:**
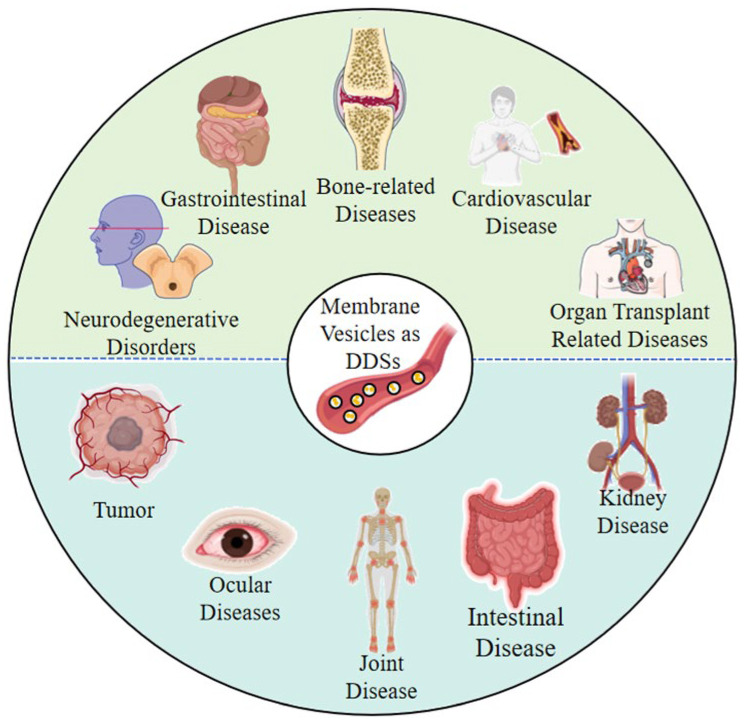
**The types of diseases that can be treated using membrane vesicles as DDSs.** The upper part (green) shows the types of diseases covered in the first edition of this Special Issue, and the lower part (blue) shows the types of diseases covered in the second edition of this Special Issue.

Membrane vesicles were initially regarded as “waste” from cellular metabolism or “by-products” secreted by bacteria. As research progressed, their unique natural advantages were discovered, such as natural delivery structures, good biocompatibility, and natural targeting potential. These advantages form the basis for their use as carriers, and research on membrane vesicles as drug carriers has thus flourished. The first edition of this Special Issue included reports on the therapeutic application of membrane vesicles as DDSs in various diseases, including bone-related, gastrointestinal, organ transplant-related, cardiovascular, and neurodegenerative diseases/disorders, among others [17] (Figure 3). The present edition of the Special Issue reports on the therapeutic application of membrane vesicles as DDSs in other diseases, including tumors [1,26,28,29] and joint [30,31], ocular [32], intestinal [27], and kidney diseases [33] (Figure 3).

An et al. [1] discussed some recent developments in the application of cell membrane vesicles to tumors, including gene editing, hybridization of cell vesicles, stimulating vesicles to produce natural anti-tumor substances before vesicle extraction, and using vesicles as drug delivery carriers, among others. Chen et al. [22] further verified the in vivo targeted delivery function of EVs by fluorescently tracing small extracellular vesicles in a human gastric cancer xenograft model. Muttiah et al. [28] summarized the application potential of extracellular EVs in breast cancer treatment, highlighting that EVs play a key role in breast cancer progression by participating in angiogenesis, immune modulation, and the formation of pre-metastatic niches. Papadakos et al. [29] discussed the unique composition of EVs, which is rich in HCC-specific biomolecules, and suggested that unlocking the full therapeutic potential of EV release in HCC could lead to transformative strategies against this challenging cancer. Yuan et al. summarized the role of EVs in the treatment of osteoarthritis (OA), mainly including immune regulation, promoting the survival of chondrocytes and facilitating the synthesis of the extracellular matrix (ECM) [30]. In the treatment of osteoarthritis, vesicle drug delivery can precisely deliver drugs to the lesion sites of the joint, effectively alleviate inflammatory responses, inhibit chondrocyte apoptosis and degradation, and promote cartilage repair [31]. The research conducted by Tian et al. [32] indicates that in the treatment of eye diseases, the vesicle drug delivery system can break through the barrier between the blood and the eye and precisely deliver drugs to the affected areas such as the retina and cornea, thereby enhancing the bioavailability of the drugs and improving their therapeutic effect. MVs as drug delivery carriers with potential clinical applications have demonstrated multi-mechanism synergistic therapeutic advantages in the treatment of inflammatory bowel disease (IBD). They can effectively inhibit key inflammatory pathways, alleviate oxidative stress, enhance intestinal barrier function, and regulate the balance of intestinal flora [27]. The research conducted by Ceccotti et al. [33] revealed that the therapeutic effect of MVs is mainly achieved by delivering miRNAs (such as miR-125b-5p and miR-146a-5p), which can effectively inhibit inflammation and fibrosis processes, block key signaling pathways such as NF-kB and STAT3, and promote the repair of renal tubular epithelial cells and angiogenesis.

Membrane vesicles, as a drug delivery system, rely on their biomimetic membrane structure and biocompatibility to precisely deliver drugs to the target site, thereby enhancing therapeutic efficacy and reducing toxic side effects. The articles in this Special Issue fully demonstrate the advantages of membrane vesicles as drug carriers in disease treatment. However, the drug-loading system of membrane vesicles still faces challenges such as insufficient drug loading efficiency, weak tissue targeting and penetration, and poor stability in the body. It still has a long way to go before it can meet clinical requirements.
